# Association between PM_10_, PM_2.5_, NO_2_, O_3_ and self-reported diabetes in Italy: A cross-sectional, ecological study

**DOI:** 10.1371/journal.pone.0191112

**Published:** 2018-01-17

**Authors:** Riccardo Orioli, Giuseppe Cremona, Luisella Ciancarella, Angelo G. Solimini

**Affiliations:** 1 Department of Public Health and Infectious Diseases, Sapienza University of Rome, Rome, Italy; 2 Sustainable Territorial and Production Systems Department, National Agency for New Technologies, Energy and Sustainable Economic Development (ENEA), Bologna, Italy; Utah State University, UNITED STATES

## Abstract

**Introduction:**

Air pollution represents a serious threat to health on a global scale, being responsible for a large portion of the global burden of disease from environmental factors. Current evidence about the association between air pollution exposure and Diabetes Mellitus (DM) is still controversial. We aimed to evaluate the association between area-level ambient air pollution and self-reported DM in a large population sample in Italy.

**Materials and methods:**

We extracted information about self-reported and physician diagnosed DM, risk factors and socio-economic status from 12 surveys conducted nationwide between 1999 and 2013. We obtained annual averaged air pollution levels for the years 2003, 2005, 2007 and 2010 from the AMS-MINNI national integrated model, which simulates the dispersion and transformation of pollutants. The original maps, with a resolution of 4 x 4 km^2^, were normalized and aggregated at the municipality class of each Italian region, in order to match the survey data. We fit logistic regression models with a hierarchical structure to estimate the relationship between PM_10_, PM_2.5_, NO_2_ and O_3_ four-years mean levels and the risk of being affected by DM.

**Results:**

We included 376,157 individuals aged more than 45 years. There were 39,969 cases of DM, with an average regional prevalence of 9.8% and a positive geographical North-to-South gradient, opposite to that of pollutants’ concentrations. For each 10 μg/m^3^ increase, the resulting ORs were 1.04 (95% CI 1.01–1.07) for PM_10_, 1.04 (95% CI 1.02–1.07) for PM_2.5_, 1.03 (95% CI 1.01–1.05) for NO_2_ and 1.06 (95% CI 1.01–1.11) for O_3_, after accounting for relevant individual risk factors. The associations were robust to adjustment for other pollutants in two-pollutant models tested (ozone plus each other pollutant).

**Conclusions:**

We observed a significant positive association between each examined pollutant and prevalent DM. Risk estimates were consistent with current evidence, and robust to sensitivity analysis. Our study adds evidence about the effects of air pollution on diabetes and suggests a possible role of ozone as an independent factor associated with the development of DM. Such relationship is of great interest for public health and deserves further investigation.

## Introduction

Air pollution is ranked high in the burden of disease attributed to environmental factors, accounting for 3.1 million deaths in 2012 and for 3.1% (2.7–3.4) of global DALYs [1]. It represents a serious and growing threat to health on a global scale involving both developed and developing countries, many of which are experiencing a fast economic growth that is often associated with the emission of huge amounts of pollutants in the environment. It’s association with several chronic conditions such as cardiovascular diseases [2,3], asthma [4,5], chronic obstructive pulmonary disease (COPD) [6,7], and cancer [8] is well documented in scientific literature.

Diabetes mellitus, for its part, represents a leading cause of morbidity and mortality among noncommunicable diseases: its global prevalence has risen to 8.5% among adults and it caused 1.5 million deaths in 2012 [9]. It has been recognized as one of four priority diseases by world leaders in the 2011 Political Declaration on the Prevention and Control of noncommunicable diseases [10]. Hence, the possibility to target air pollution as a modifiable risk factor on which to take action for reducing the diabetes epidemic is of great interest for public health, especially considering its wide diffusion in the population.

The biological plausibility of such relationship is supported by several toxicological studies. Particulate matter was found to activate different inflammatory pathways leading to endothelial dysfunction and insulin resistance [11,12]. It was demonstrated also to induce metabolic impairment, increasing adiposity and inflammation in brown and white adipose tissue. The exposure to ozone was associated with altered glucose and lipid metabolism, activating stress-hormones responses [13,14].

Epidemiological evidence is more controversial. The first ecological study suggesting an association between diabetes mellitus and ambient air pollution was published by Lockwood in 2002 [15]. He found that diabetes prevalence was significantly correlated with total annual air emissions at state-level in the US. More recently, Pearson et al. observed a 1% increase in diabetes prevalence per 10 μg/m^3^ increase in PM_2.5_ levels among US counties [16]. Another ecological study conducted in Italy at the province level found an association between PM_2.5_ levels and diabetes-linked hospital discharges, after adjusting for known risk factors and an indicator of appropriateness in hospitalizations [17]. Evidence coming from longitudinal studies is increasing and it has been synthesized in many meta-analyses [18–20], but the results are still controversial. In particular, there is paucity of evidence about the relationship between exposure to ozone and diabetes. To our knowledge, only one cohort study explored the effect of ozone on the development of T2DM, reporting a significant effect [21]. However, it was limited to the specific population subgroup of African American women.

The purpose of this study was to evaluate the ecological association between area-level air pollution (PM_10_, PM_2.5_, NO_2_, and O_3_) and self-reported diabetes mellitus in a large, nationwide Italian population sample. Furthermore, we explored effect modification in order to detect possible variations in susceptibility among different subgroups and to identify the characteristics of more susceptible individuals.

Our hypothesis was that an increased level of exposure to air pollution is associated with an increase in prevalent diabetes, when accounting for important individual covariates.

## Methods

### Study population

We collected data regarding diabetes and the covariates from twelve National surveys, regularly conducted by ISTAT (Italian National Institute of Statistics) from 1999 to 2013. In particular we used data from the available version of Multipurpose National Surveys on households about “Health conditions and use of medical services”, carried out in 1999–2000, 2004–2005 and 2012–2013, as well as those of nine National Surveys on households about “Aspects of daily life”, carried out annually from 2003 to 2012 (except for 2004). These are population-based cross-sectional surveys that investigate many aspects about individual health status, socio-economic status, interactions with social and health services, and daily life activities. They share a complex study design, characterized by a stratified multistage cluster sampling, to get reliable prevalence estimates at regional and municipality-class level (see the “Air pollution” paragraph below), as well as many questionnaire’s items, as described in detail elsewhere [22,23]. From all the available items, we selected those relevant for the outcome and the covariates of interest. We merged the single survey datasets, assembling a study population of 826,080 subjects sampled from all regions and municipality classes. Of the total, we included 376,157 individuals aged over 45.

### Outcome and covariates

We considered diabetic the subjects who reported a physician-diagnosed diabetes mellitus. We selected covariates based on well known diabetes’ risk factors and according to current evidence. They included sex, age, BMI (continuous, derived from height and weight), physical activity (binomial, yes = subjects who declared to practice sport activity or reporting a moderate to intense physical activity in leisure time or in their working setting), smoking status (never smoker, current smoker or former smoker), presence of comorbid cardiovascular conditions (binomial, yes = subjects who declared to be affected by hypertension, angina, or ischemic heart disease) and socioeconomic status, which was evaluated through questions about educational level (categorical, low = no education to lower secondary school; intermediate = high school degree; high = any university degree after high school), occupational status (categorical: employed, unemployed, housewife, retired, other condition), perceived household income (binomial, high = subjects who evaluated their household income as high or very high; low = subjects who evaluated their household income as low or insufficient), and marital status (categorical: single, married, separated/divorced, widowed).

### Air pollution

We used air pollution levels at 4 x 4 km^2^ horizontal spatial resolution estimated with the AMS Model, the Italian integrated Assessment Modelling System developed in the MINNI project funded by Italian Ministry of Environment for supporting the international negotiation process on air pollution and assessing air quality policies at national/regional level [24,25]. The core of the AMS-MINNI modelling system is the three-dimensional Eulerian model Flexible Air Quality Regional Model (FARM) [26,27], that includes transport and multiphase chemistry of pollutants in the atmosphere. The AMS-MINNI outputs are systematically compared with measured concentrations of rural, urban and suburban background air quality monitoring stations, showing (on NO_2_, PM_10_ and O_3_) good performances, according to the statistical indicators currently used in air quality models performance assessment [28,29].

For PM_10_, PM_2.5_, and NO_2_, we calculated the annual mean concentrations using AMS-MINNI model hourly simulated data. For Ozone, we used daily maximum of 8-hours running average, considering only the warm period lasting from April to September. Data were available for years 2003, 2005, 2007 and 2010.

For the exposure assessment, we had to match the gridded data with the administrative reference level allowed by the study population dataset from ISTAT surveys; to do that, we upscaled the single cell concentration levels to the administrative unit and then calculated mean, median and 90th percentile annual values for each pollutant (April-September for Ozone). We started from the resolution of 4 x 4 km^2^, and using overlay GIS-based procedures and the weighted mean method, we firstly recalculated the dataset at municipal level (pollutant-specific maps at municipal level are shown in **S1–S4 Figs**) and then at the aggregation level. For the second step, we used the resident population of each municipality from Italian National Census 2011 to weight the attributed concentrations within their reference group. The aggregation level was defined by the combination of region of residence and a six-level categorical classification of municipalities based on the number of resident inhabitants (0–2,000; 2,001–10,000; 10,001–50,000; more than 50,000; regional capitals; municipalities within regional capital metropolitan areas). This classification, although approximate, showed good homogeneity of air quality for municipalities belonging to the same group. Finally, we matched the two datasets by the combination of these two variables and attributed to each individual the corresponding levels of exposure to air pollutants.

### Statistical analysis

We firstly carried out descriptive analysis to assess population sample characteristics, geographical distribution and correlation coefficients between variables. The binary diabetes outcome was regressed in a mixed logistic model against the mean levels of each pollutant, controlling for age, sex, BMI, educational level, occupational status, marital status, physical activity, household income, and smoking status as fixed variance components. We selected survey-identifying code and region of residence as random variance components, in order to deal with the hierarchical structure of the data. We included in covariates also the geographic coordinates for each regional capital (latitude and longitude) to have some adjustment for spatial autocorrelation. We centred on the mean all numerical variables (age, BMI and pollutant levels) to limit the effect of different units of measurement.

We used a stepwise regression approach to evaluate the effect of adding-in of single covariates on on goodness of fit, assessed through Akaike Information Criterion (AIC), with lower AIC values indicating a better fit. To assess possible multicollinearity between variables, we computed the variance inflation factor (VIF).

We tested for effect modification by gender, age class (<60, 60–74, ≥75), weight status (normal/underweight, overweight, obese), physical activity (yes, no), smoking status (never smoker, current smoker, former smoker) and educational level (high, intermediate, low). We also explored possible effect modification due to the presence of comorbid cardiovascular diseases (hypertension, angina, ischemic heart disease). We generated interaction terms for each pollutant with the potential effect modifier, and added them one by one to the fully adjusted model. Then, we assessed heterogeneity through the likelihood ratio test and recorded the respective p-values.

For sensitivity analysis, we used the same models with different exposure metrics (utilizing annual median and 90° percentile values, or IQR as units of increase). Additionally, we added the mean-centred proportion of diabetic people for each geographical area, to have some contextual adjustment for possible area-level residual confounding. We also evaluated the effect of air pollutants in the subgroup of the population with exposure levels beyond the limits recommended by WHO [30]. Finally, we performed two-pollutant models for pairs of pollutants. We only tested for pairs formed by ozone and each other pollutant due to high correlation between PM_10_, PM_2.5_ and NO_2_.

We explored the shape of relationships between exposures and outcome by replacing the linear term in the fully adjusted models with B-splines with 3 degrees of freedom (df) and compared the goodness of fit through AIC and likelihood-ratio test.

All statistical analyses were carried out with R version 3.2.3 (The R Foundation for Statistical Computing, Vienna, Austria), using the packages lme4 for mixed models and dlnm for non-linear approach and plots. We considered as indication of statistical significance p-values equal or less than 0.05.

## Results

Descriptive statistics of the population sample by diabetic status are reported in **Table 1**. Mean (SD) age was 63.5 (11.8); 53.7% of subjects were female, 13.9% were obese, 17.9% were smokers, 66.8% had a secondary school educational level or lower and 38.2% reported low family income. In total 36,969 individuals reported a diagnosis of diabetes, with an overall prevalence of 9.8%. Compared to females, males had lower mean age, were more likely obese or overweight, current or ex-smokers and had a slightly higher family income and educational level. Diabetic people were more likely to be female, current or previous smokers, had a lower family income and educational level and a higher mean age and BMI, compared to the entire population sample. The mean (SD) exposure in the study population was 16.9 μg/m^3^ (7.4) for PM_10_, 15.9 μg/m^3^ (7.1) for PM_2.5_, 15.9 μg/m^3^ (11.3) for NO_2_, and 103.2 μg/m^3^ (5.1) for O_3_.

**Table 1:** Descriptive statistics of the study population by diabetic status.

Characteristic n (%)	Non diabetic 339,188	Diabetic 36,969	All 376,157
**Sex**			
Males	156,880 (46.3)	17,186 (46.5)	174,066 (46.3)
Females	182,308 (53.7)	19,783 (53.5)	202,091 (53.7)
**Age class**			
<60	153,112 (45.1)	6,897 (18.7)	160,009 (42.5)
60–74	123,009 (36.3)	16,729 (45.3)	139,738 (37.1)
≥75	63,067 (18.6)	13,343 (36.1)	76,410 (20.3)
**Weight status**			
Normal/Underweight	155,706 (45.9)	11,538 (31.2)	167,244 (44.5)
Overweigh	140,367 (41.4)	16,390 (44.3)	156,757 (41.7)
Obese	43,115 (12.7)	9,041 (24.5)	52,156 (13.9)
**Physical activity**			
Yes	119,244 (35.2)	7,687 (20.8)	126,931 (33.7)
No	219,944 (64.8)	29,282 (79.2)	249,226 (66.3)
**Smoking status**			
Never smoker	176,693 (52.1)	20,101 (54.4)	196,794 (52.3)
Former smoker	96,433 (28.4)	12,238 (33.1)	108,671 (28.9)
Current smoker	62,941 (18.6)	4,433 (12)	67,374 (17.9)
**Educational level**			
High	35,925 (10.6)	1,779 (4.8)	37,704 (10)
Intermediate	81,661 (24.1)	5,600 (15.1)	87,261 (23.2)
Low	221,602 (65.3)	29,590 (80)	251,192 (66.8)
**Occupational status**			
Employed	109,784 (32.4)	4,458 (12.1)	114,242 (30.4)
Unemployed	8,894 (2.6)	618 (1.7)	9,512 (2.5)
Housewife	69,664 (20.5)	8,451 (22.9)	78,115 (20.8)
Retired	134,026 (39.5)	20,187 (54.6)	154,213 (41)
Other	16,820 (5)	3,255 (8.8)	20,075 (5.3)
**Household income**			
High	211,162 (62.3)	19,226 (52)	230,388 (61.2)
Low	126,280 (37.2)	17,582 (47.6)	143,862 (38.2)
**Marital status**			
Single	28,334 (8.4)	2,368 (6.4)	30,702 (8.2)
Married	235,179 (63.3)	22,762 (61.6)	257,941 (68.6)
Separated/divorced	21,178 (6.2)	1,489 (4)	22,667 (6)
Widowed	54,497 (16.1)	10,350 (28)	64,847 (17.2)
**CVD**			
No	230,884 (68.1)	13,804 (37.3)	244,688 (65)
Yes	108,304 (31.9)	23,165 (62.7)	131,469 (35)
**Age**			
mean (SD)	62.8 (11.7)	70.0 (10.7)	63.5 (11.8)
**BMI**			
mean (SD)	25.7 (3.9)	27.4 (4.4)	25.9 (4.0)
**Estimated exposure to** **air pollutants [mean (SD)]**			
PM_10_	16.9 (7,4)	16.4 (7,1)	16.9 (7,4)
PM_2,5_	15.9 (7.1)	15.4 (6.8)	15.9 (7.1)
NO_2_	16.0 (11.3)	15.2 (11.1)	15.9 (11.3)
O_3_	103.2 (5.1)	103.0 (4.8)	103.2 (5.1)

**Table 2** shows descriptive statistics of PM_10_, PM_2.5_, NO_2_ and O_3_ variables based on the municipalities classification. As expected, pollutants levels were higher in regional capitals, municipalities within regional capital metropolitan areas and more densely populated municipalities. Pollutants’ correlation matrix showed Pearson’s coefficients about 0.30–0.40 for all the combinations that included O_3_, while for combinations of particulate matter and NO_2_ the coefficients were very high (>0.90 between PM_10_ and PM_2.5_, and between PMs and NO_2_), confirming the chemical transformations in AMS-MINNI model and the strong weight of NO_2_ precursor in secondary component of PM. The high correlation is probably also due to the aggregation process, which tend to reduce the differences among correlated pollutants.

**Table 2:** Descriptive statistics of the spatial variation of PM_10_, PM_2.5_, NO_2_ and O_3_, according to the municipality class.

Municipality class	PM_10_ (μg/m^3^)		PM_2.5_ (μg/m^3^)		NO_2_ (μg/m^3^)		O_3_ (μg/m^3^)	
	Median	Min-Max	Median	Min-Max	Median	Min-Max	Median	Min-Max
Class 1	23.7	14.2–52.6	22.3	12.7–49.7	28.4	15.5–64.8	106.3	94.8–114.1
Class 2	16.8	10.7–37.2	15.9	9.6–35.3	17.2	8.2–47.9	106.4	100.0–112.7
Class 6	15.6	10.7–31.2	14.8	9.6–29.6	15.0	4.0–38.2	104.5	93.5–114.8
Class 5	13.5	9.8–28.0	12.6	8.7–26.5	12.6	3.4–32.8	103.1	86.5–114.6
Class 4	12.0	6.5–23.4	11.3	6.1–22.3	7.8	2.7–25.5	101.2	86.2–113.5
Class 3	10.7	6.0–18.2	10.1	5.6–17.4	5.2	1.9–15.5	99.8	85.5–110.1

Class 1: most populated regional capitals (Turin, Milan, Venice, Genoa, Bologna, Florence, Rome, Naples, Bari, Cagliari, Palermo, Catania); Class 2: municipalities within regional capital metropolitan areas; Class 3: municipalities with less than 2,000 resident inhabitants; Class 4: municipalities with 2,001–10,000 resident inhabitants; Class 5: municipalities with 10,001–50,000 resident inhabitants; Class 6: municipalities with more than 50,000 resident inhabitants.

The classes are ordered by decreasing air pollutants concentration levels.

We observed a positive and significant association between the mean levels of all pollutants and the probability of being affected by diabetes, expressed as Odds Ratio (OR) per increase of 10 μg/m^3^ [PM_10_ 1.04 (95% CI 1.01–1.07), PM_2.5_ 1.04 (95% CI 1.02–1.07), NO_2_ 1.03 (95% CI 1.01–1.05), O_3_ 1.06 (95% CI 1.01–1.11)] (**Table 3**).

**Table 3:** Association between air pollution and diabetes mellitus by different exposure metrics and sensitivity analysis.

Sensitivity analysis	PM_10_ OR (95% CI)	PM_2.5_ OR (95% CI)	NO_2_ OR (95% CI)	O_3_ OR (95% CI)
Unadjusted logistic regression (mean values; 10 μg/m^3^ increase)	0.90 (0.89–0.92)	0.89 (0.88–0.91)	0.94 (0.93–0.95)	0.93 (0.91–0.95)
**Main mixed model** (mean values; 10 μg/m^3^ increase)	1.04 (1.01–1.07)	1.04 (1.02–1.07)	1.03 (1.01–1.05)	1.06 (1.01–1.11)
median values (10 μg/m^3^ increase)	1.05 (1.02–1.08)	1.06 (1.02–1.09)	1.03 (1.01–1.05)	1.07 (1.02–1.11)
90^th^ percentile values (10 μg/m^3^ increase)	1.02 (1.01–1.03)	1.02 (1.01–1.03)	1.02 (1.01–1.03)	1.06 (1.03–1.09)
IQR increase*	1.04 (1.02–1.08)	1.05 (1.02–1.08)	1.08 (1.06–1.09)	1.04 (1.01–1.06)
main model + diabetes prevalence	1.05 (1.03–1.07)	1.05 (1.03–1.08)	1.04 (1.03–1.05)	1.07 (1.03–1.12)
below WHO limits**	1.26 (1.17–1.35)	2.56 (1.79–3.66)	1.06 (1.04–1.09)	1.33 (1.15–1.54)
two pollutants models	1.04 (1.01–1.06)	1.04 (1.01–1.07)	1.03 (1.01–1.04)	/
O_3_	1.05 (1.01–1.10)	1.05 (1.01–1.10)	1.05 (1.00–1.10)	/

* PM_10_: 8.9 μg/m^3^; PM_2.5_: 9.2 μg/m^3^; NO_2_: 14.7 μg/m^3^; O_3_: 6.8 μg/m^3^.

** According to WHO Air Quality Guidelines (2006) recommended annual average levels. PM_10_: 20 μg/m^3^; PM_2.5_: 10 μg/m^3^; NO_2_: 40 μg/m^3^; O_3_: 100 μg/m^3^.

Results of the stepwise regression approach are provided separately in **S1 Table**. They indicated that maximum goodness of fit was reached by the fully adjusted models. According to Variance Inflation Factor (VIF), there was no evidence of multicollinearity between variables in our fully adjusted models (all values <5).

**Table 4** shows the results of effect modification analysis. For PM_10_ and PM_2.5_, we observed effect modification by gender, smoking status, presence of CVD and household income (0.05 for PM_10_). For NO_2_, the interaction with gender, smoking status and household income were significant. For O_3_, we found evidence of effect modification by gender and household income, and marginally non-significant interactions with physical activity. Where significant, there were enhanced effects among men, current or former smokers, individuals not affected by CVD and with low household income. Additionally, we found enhanced effects among the elderly (≥75 years old), obese, physically inactive and those with low educational level, but p-values deriving from likelihood ratio test were non-significant.

**Table 4:** Modification of the associations* between air pollutants (for 10 μg/m^3^ increase) and diabetes mellitus by characteristics or presence of comorbid conditions.

Characteristics	N diabetic/N tot	PM_10_ OR (95% CI)	PM_2.5_ OR (95% CI)	NO_2_ OR (95% CI)	O_3_ OR (95% CI)
**Sex**					
Males	17,186/174,066	**1.09 (1.05–1.12)**	**1.09 (1.06–1.13)**	**1.06 (1.04–1.08)**	**1.10 (1.04–1.16)**
Females	19,783/202,091	1.00 (0.97–1.03)	1.00 (0.97–1.03)	1.00 (0.99–1.02)	1.03 (0.98–1.07)
Interaction p-value**		**<0.001**	**<0.001**	**<0.001**	**0.006**
**Age class**					
<60	6,897/160,009	1.02 (0.98–1.07)	1.03 (0.99–1.07)	1.01 (0.99–1.04)	**1.07 (1.00–1.14)**
60–74	16,729/139,738	**1.04 (1.01–1.07)**	**1.04 (1.01–1.07)**	**1.03 (1.01–1.05)**	1.03 (0.98–1.09)
≥75	13,343/76,410	**1.04 (1.00–1.07)**	**1.04 (1.00–1.08)**	**1.03 (1.01–1.05)**	**1.09 (1.03–1.15)**
Interaction p-value**		0.795	0.868	0.455	0.120
**BMI**					
Normal/Underweight	11,538/167,244	1.02 (0.98–1.05)	1.02 (0.98–1.06)	1.02 (1.00–1.04)	1.02 (0.97–1.08)
Overweigh	16,390/156,757	**1.05 (1.02–1.08)**	**1.06 (1.02–1.09)**	**1.03 (1.01–1.05)**	**1.08 (1.02–1.14)**
Obese	9,041/52,156	**1.06 (1.01–1.11)**	**1.07 (1.02–1.12)**	**1.04 (1.01–1.07)**	**1.10 (1.02–1.19)**
Interaction p-value**		0.184	0.155	0.437	0.194
**Physical activity**					
Yes	29,282/249,226	1.03 (0.99–1.07)	1.04 (0.99–1.08)	1.02 (0.99–1.04)	1.02 (0.96–1.09)
No	7,687/126,931	**1.04 (1.01–1.07)**	**1.04 (1.02–1.07)**	**1.03 (1.02–1.05)**	**1.07 (1.02–1.12)**
Interaction p-value**		0.681	0.701	0.303	0.083
**Smoking status**					
Never smoker	20,101/196,794	1.01 (0.98–1.04)	1.01 (0.98–1.04)	1.02 (1.00–1.04)	1.04 (0.99–1.09)
Former smoker	12,238/108,671	**1.05 (1.02–1.09)**	**1.06 (1.02–1.09)**	**1.04 (1.02–1.06)**	**1.09 (1.03–1.16)**
Current smoker	4,433/67,374	**1.10 (1.05–1.15)**	**1.11 (1.06–1.16)**	**1.06 (1.03–1.09)**	1.07 (0.99–1.16)
Interaction p-value**		**<0.001**	**<0.001**	**0.014**	0.164
**Educational level**					
High	1,779/37,704	1.00 (0.94–1.07)	1.00 (0.94–1.07)	1.00 (0.96–1.04)	1.02 (0.91–1.14)
Intermediate	5,600/87,261	1.04 (1.00–1.08)	1.04 (1.00–1.09)	1.02 (1.00–1.05)	1.06 (0.99–1.14)
Low	29,590/251,192	**1.05 (1.02–1.07)**	**1.05 (1.02–1.08)**	**1.04 (1.02–1.05)**	**1.06 (1.01–1.11)**
Interaction p-value**		0.432	0.425	0.211	0.792
**Household income**					
High	19,226/230,388	1.02 (0.99–1.05)	1.03 (0.99–1.06)	**1.02 (1.00–1.04)**	1.04 (0.99–1.09)
Low	17,582/143,862	**1.06 (1.03–1.09)**	**1.06 (1.03–1.10)**	**1.04 (1.02–1.06)**	**1.09 (1.04–1.15)**
Interaction p-value**		0.055	**0.043**	**0.035**	**0.031**
**CVD**					
No	13,804/244,688	**1.06 (1.03–1.09)**	**1.06 (1.03–1.10)**	**1.04 (1.02–1.06)**	**1.06 (1.01–1.12)**
Yes	23,165/131,469	1.03 (1.00–1.06)	1.03 (1.00–1.06)	**1.02 (1.01–1.04)**	**1.06 (1.01–1.11)**
Interaction p-value**		0.055	**0.041**	0.161	0.925

* Adjusted for: age, sex, BMI, physical activity, smoking status, educational level, marital status, occupational status, and household income, except when tested as effect modifier; survey and region of residence as random variance components.

** From likelihood-ratio test for interaction.

Statistically significant results are in **bold**.

In sensitivity analysis (**Table 3**), the use of median and 90^th^ percentile as metrics of exposure to pollutants resulted in associations with similar magnitude and strength, as well as the use of IQR as unit of increase. Adding diabetes prevalence as a contextual variable caused a slight increase in the strength of the association, which remained of similar magnitude. The associations were robust to adjustment for other pollutants in all combination tested in two pollutant models (PM_10_ + O_3_; PM_2.5_ + O_3_; NO_2_ + O_3_). In the population subgroup with exposure levels below WHO air quality guidelines, we observed a remarkable increase in the magnitude of the association for PM_10_ [1.26 (95% CI 1.17–1.35)], PM_2.5_ [2.56 (95% CI 1.79–3.66)], and O_3_ [1.33 (95% CI 1.15–1.54)], and a slight increase for NO_2_ [1.06 (95% CI 1.04–1.09)].

**Fig 1:**
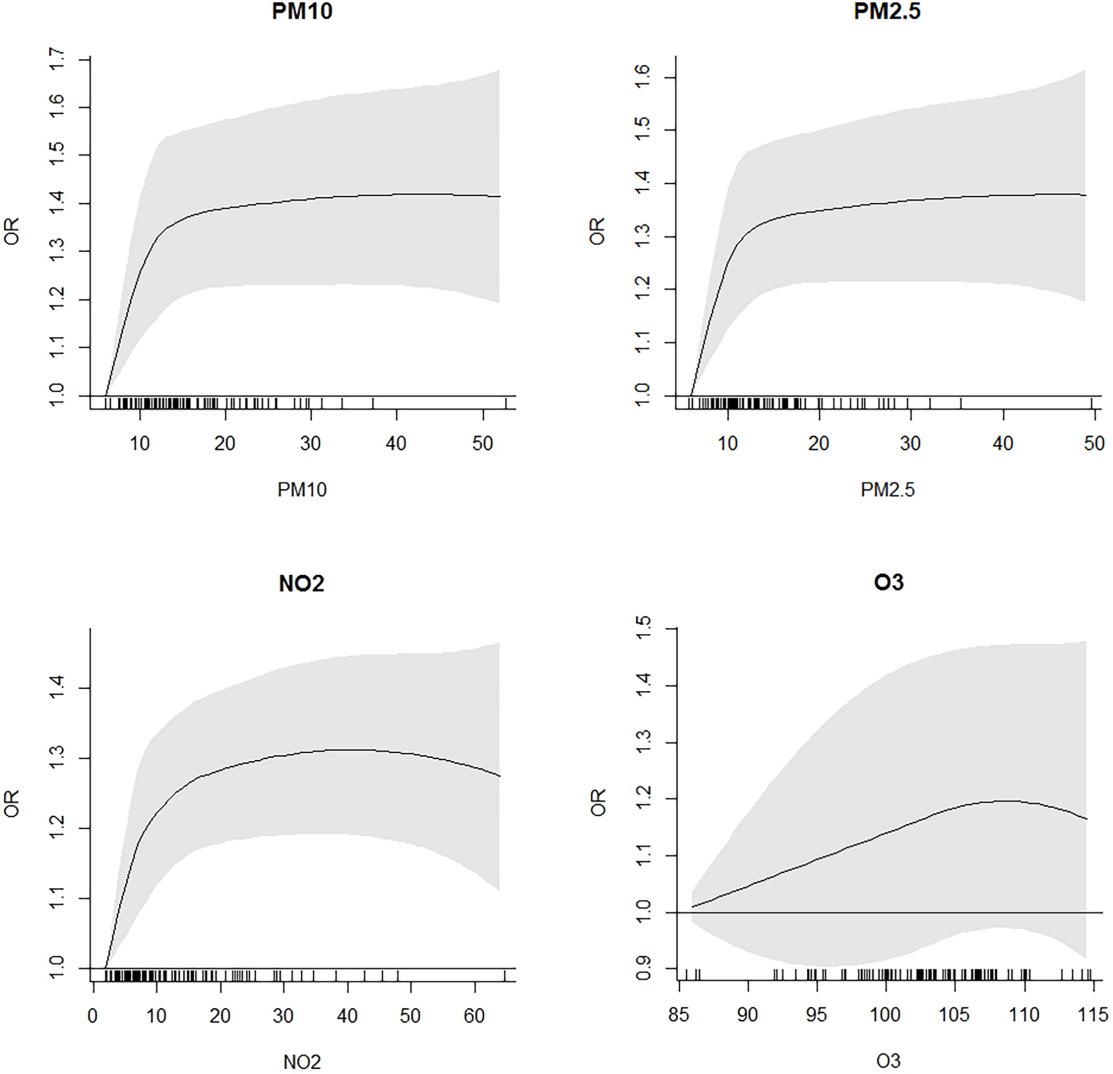
Estimated concentration-response curves and 95% CIs for pollutants (PM_10_, PM_2.5_, NO_2_, and O_3_) and diabetes mellitus.

According to this results, indicating a possible non-linear exposure-outcome relationship, we used a non-parametric approach and introduced a B-spline with 3 degrees of freedom instead of the linear term for each pollutant. **Fig 1** shows the estimated concentration-response curves, while comparison between models are shown in **Table 5**. We observed a steep almost linear relationship at low concentrations for PM_10_, PM_2.5_ and NO_2_, and then a progressive reduction and a plateau effect, although higher levels had few observations. Goodness of fit tests indicating a better fit for non-linear models. Differently, the association with ozone didn’t seem to deviate from linearity except for higher concentrations, which had few observations too; the introduction of the spline term did not improve the performance of the model.

**Table 5:** Comparison between standard fully adjusted models and non-linear models, according to goodness of fit.

Sensitivity analysis	PM_10_	PM_2.5_	NO_2_	O_3_
**Linear model AIC**	212,475	212,475	212,470	212,477
**Spline model AIC**	212,455	212,454	212,446	212,479
p-value*	**<0.001**	**<0.001**	**<0.001**	0.341

*from likelihood ratio test

## Discussion

In our study, the risk of being affected by diabetes was significantly associated with estimated long-term exposure to PM_10_, PM_2.5_, NO_2_ and O_3_ in the area of residence, after controlling for important individual covariates. This is the first ecological study conducted nationwide in Italy to explore the relationship between type 2 diabetes mellitus and those four pollutants at the same time. Nowadays there is still a steep socio-economic North to South gradient that reflects on the prevalence of diseases related to socio-economic factors [31]. Hence, diabetes geographical pattern in Italy tend to be opposite to the pollutant’s one, as the most developed, urbanized and industrialized areas are located in the Northern part of the country, while the most important risk factors for DM are more prevalent in South and Islands. It is not surprising that crude logistic regressions resulted in negative associations (**Table 3**) until we used a hierarchical approach by including region and survey year as random components.

Evidences on the association between air pollution and diabetes mellitus deriving from longitudinal studies are increasing, but still mixed [32–37]. Some studies found associations only among specific subgroups [38–42]. However, even available meta-analyses supported the existence of an association, reporting coefficients similar to or slightly higher than ours [18–20]. In general, our results were more similar to those of the studies with broad population samples [32,40,42], although it is necessary to take into account the differences in exposure assessment, as well as in measured covariates and study design. Moreover, differently from our study, geographical pattern of diabetes and air pollution tended to be concordant (e.g. positive spatial correlation) in the other study areas. Even if we could rely on detailed individual information and we modeled the associations accounting for geographic characteristics, we cannot exclude the presence of residual confounding from area-level factors accounting for the differences in the effect size. However such effect, if present, is probably small as adding contextual diabetes prevalence to the main model changed the magnitude of the association with air pollutants by ~1%.

In our study, ozone showed a different geographical pattern compared to particulate matter and NO_2_ and a low correlation with them. In most of our models, the association of ozone with prevalent diabetes was slightly higher compared to the other pollutants, but usually with lower p-values. This association remained robust also in two-pollutant models, suggesting that the effects may be independent from each other. Ozone at ground level is one of the major constituents of photochemical smog. In fact, its levels do not depend only on traffic and industry emissions, as it is formed by reaction of precursors such as nitrogen oxides (NO_x_) and volatile organic compounds (VOCs) in the presence of sunlight. As a result, the highest levels of ozone pollution occur in areas with more solar radiation and in periods of sunny weather [30]. However, the association between O_3_ and diabetes is still understudied. Most of the epidemiological studies about ozone impact on health deal with short- and long-term respiratory and cardiovascular effects or with all cause and cause-specific mortality. To our knowledge, there is only one study on the association between long-term exposure to ozone and incident diabetes available to date [21]. This is a prospective study conducted nationwide in the US on a cohort of 45,231 African American women. The HR reported by authors for incident diabetes was 1.18 (95% CI 1.04–1.34) per IQR increment of O_3_ (6.7 ppb, about 13 μg/m^3^). Although effect size is higher than our, this study focuses on a specific population subgroup that has been demonstrated to be at higher risk of diabetes [43] and an enhanced susceptibility of black women to the effects of ozone compared to other ethnicities cannot be excluded. The initial but limited evidence deriving from our results and previous studies suggests that the impact of long term exposure to ozone on diabetes mellitus may be relevant for public health policies and should be further investigated, especially since many regions worldwide are experiencing increases in ozone levels, expected to grow even more in the future with the effects of climate change.

The results of effect modification analysis showed stronger associations among men compared to women (Table 3). This is not unprecedented, as another study had similar findings, although not significant [33]. However, the majority of previous studies found stronger effects among women. A 2008 study on patients attending two respiratory health clinics in Canada showed a relationship between modeled NO_2_-concentration and the prevalence of diabetes mellitus among women, but not among men, with an OR of 1.04 (1.00–1.08) per 1 ppb increase [38]. Puett et al. observed an association with distance to road (<50 m vs ≥200 m from a roadway) among women, while no evidence was found among men [42]. Such difference was mainly attributed by authors to the exposure assessment adopted, based on home addresses, as women tend to spend more time at home compared to men. Our exposure assessment is probably less affected by this problem, being based on a municipality level resolution, and may be more representative of the exposure level of individuals that spend more time away from home. Furthermore, adding gender-restricted diabetes prevalence in covariates resulted in a significant association even in women, except for ozone (data not shown). Although gender-related differences in predisposition to the effects of air pollution cannot be excluded, this result may also be due to the effect of residual area-level confounding. We also found stronger effects among current or former smokers compared to never smokers. This is coherent with the toxicological mechanism invoked in the association between air pollution and diabetes, such as vascular inflammation and atherosclerosis, which are involved in smoke effects on cardiovascular diseases too. Interestingly, we observed stronger effects of PM_10_ and PM_2.5_ among individuals not affected by other cardiovascular diseases. Other studies had similar findings, but the difference was not statistically significant [32,40].

When we restricted the analysis to exposure levels below the limits recommended by WHO [30], we observed an overall increase in the strength of the associations. Such increase was remarkable for PM_2.5_, with an OR of 2.73 (95% CI 1.92–3.90, p<0.001) per 10 μg/m^3^ increase. Increased effect size at exposure levels below air quality standards have been already observed in other studies, with both diabetes [16,33,37] and other cardiovascular events [44,45] as outcome. This may imply a non-linear relationship between air pollution and diabetes. The use of a non-parametric approach, with the introduction of a spline term instead of linear term, seemed to support such hypothesis for PM_10_, PM_2.5_ and NO_2_, showing a steep linear relationship for low concentrations (about 0–12 μg/m^3^) and then a fast decrease and a plateau effect starting from concentrations around 15 μg/m^3^ (**Fig 1** and **Table 5**). However, the great majority of the observations are condensed within this range, and results observed in higher ranges are probably less reliable. Future research should address this issue trying to study populations more equally exposed to a wider range of air pollution concentrations.

The use of different annual measures for pollutants (i.e. median and 90^th^ percentile) did not alter significantly the patterns of the association in our study, as well as the use of IQR as unit of increase.

Our findings are consistent with evidence coming from mechanistic studies, which suggested different possible pathways involved in this relationship. The first mechanism proposed for particulate matter is endothelial dysfunction: in both mice and human model studies, it has been demonstrated that exposure to PM promotes vascular inflammation and atherosclerosis [11,46]. Insulin ability to induce glucose uptake is in part mediated by the regulation of the vascular tone [12], and it is likely that interferences in this mechanism could account for a propensity to insulin resistance. The connection between air quality, inflammation and insulin resistance, that is one of the most important underlying metabolic conditions predisposing to T2DM, is reported also in epidemiological studies. In a 2009 cross-sectional study conducted in Isfahan (Iran) on children and young adults, the authors observed an association between air pollution and insulin resistance (assessed through HOMA-IR), independently from BMI, physical activity and dietary intake [47]. Another mechanism that seem to be implicated is the development of alterations in visceral adipose tissue, with inflammation and increased adiposity eliciting insulin resistance [48]. Eze et al. found that a common interleukine-6 gene polymorphism was an effect modifier of the association between long-term exposure to PM_10_ and DM in their Swiss cohort, adding further evidence to the inflammatory pathways hypothesis [49]. Other possible mechanisms included hepatic altered insulin signaling, endoplasmic reticulum stress and mitochondrial dysfunction [50]. Other studies have also shown that T2DM, metabolic syndrome and other conditions increase the pro-inflammatory effects of air pollution [51]. Ozone was found to induce impaired glucose, lipid and amino acid metabolism, insulin resistance and oxidative stress in rats [52], and to provoke similar stress-hormones response and metabolic alterations in humans [14]. However, studies with inconsistent findings exist [53,54], and most of the mechanistic pathways involved in this association remain unclear, due to both the multifactorial pathogenesis of diabetes and the heterogeneous composition of air pollution.

An important strength of our study is that it is based on official and statistically validated data coming from nationwide surveys designed to be representative of the entire population. We could rely on a very large sample size, which is necessary when investigating such a slight association, and on detailed information about the main individual behavioral and socio-economic risk factors involved in the development of diabetes mellitus that are rarely available in similar studies. Furthermore, the estimates of diabetes prevalence calculated from the national surveys include also those individuals treated in the ambulatory and primary care and are probably more accurate than those coming from hospital discharge registries only, which underestimate the true prevalence.

This study has also several limitations. The first one regards the study design: even if the association we found was significant, this is an ecological association and it does not allow us to make any inference about causal relationships. Second, as many other ecological studies, we attributed exposure levels on a geographical basis with a low-resolution scale, which may lead to exposure misclassification. In fact, we are assuming that air pollution levels in a specific area are representative of the real long-term exposure of each individual living in that area. However, it was interesting to observe how the introduction of a hierarchical structure and of covariates in the model changed the sign and strength of the association, thus confirming how accounting for group level effects and individual-level characteristics is useful to limit ecological fallacy (**Table 3**). Another limitation is that the outcome assessment was based only on self-report of diabetes cases diagnosed by a physician. Other studies reported very little differences between self-reported and confirmed diagnoses [36]. However, this may lead to outcome overestimation in those areas were people have access to better quality healthcare services and may have higher probabilities to be diagnosed with their condition. In Italy, the regions with better healthcare performances are in the Northern or north-central areas, which are usually the most developed and, consequently, the most polluted ones. However, the choice of modeling the association including the region of residence as random effect should have accounted for this effect. No information was available on residential history, indoor and workplace exposures, commuting habits, diabetes family history, as well as on diet and alcohol consumption. In addition, socio-economic level was evaluated only through self-reported occupational status and self-perception of household income, and no objective measures were available. Furthermore, our study does not distinguish between type 1 and type 2 diabetes mellitus, although our population was aged above 44 years and T2DM have been reported to account for more than 90% of cases in adults [55].

## Conclusions

We found a significant association between self-reported diabetes mellitus and area-level annual mean levels of all examined air pollutants (PM_10_, PM_2.5_, NO_2_ and O_3_), in a large population sample in Italy. This association was robust utilizing different measures for the exposure estimate, and two pollutant models including Ozone showed independent, significant effects of each pollutant. We found significant effect modification by gender, smoking status and presence of comorbid cardiovascular conditions, with enhanced effects on males, current or former smokers and in people without other cardiovascular diseases (particulate matter only). We then observed a general increase in the strength of the association when considering exposure values below WHO recommended annual limits.

Our study contributes to the growing body of evidence that supports a role of air pollutants in the development of diabetes mellitus, although the observed relationship cannot be considered causal, due to study design. In particular, we find that the effects of long-term exposure to Ozone on diabetes are still neglected and should be further investigated.

## Supporting information

S1 Fig Map of the concentrations of PM_10_ at municipality level across Italy (AMS-MINNI). Averaged annual levels for years 2003, 2005, 2007 and 2010.
(TIF)

S2 Fig Map of the concentrations of PM_2.5_ at municipality level across Italy (AMS-MINNI). Averaged annual levels for years 2003, 2005, 2007 and 2010.
(TIF)

S3 Fig Map of the concentrations of NO_2_ at municipality level across Italy (AMS-MINNI). Averaged annual levels for years 2003, 2005, 2007 and 2010.
(TIF)

S4 Fig Map of the concentrations of O_3_ at municipality level across Italy (AMS-MINNI). Averaged annual levels for years 2003, 2005, 2007 and 2010.
(TIF)

S1 Table Stepwise regression for the main model, with forward selection of predictors. Mixed models with region and survey year as random components. AIC: Akaike Information Criterion. *from likelihood ratio test, each model tested with the preceding one.
(DOCX)

## Abbreviations

AIC: Akaike Information Criterion
AMS-MINNI: the Italian integrated Assessment Modelling System for supporting the international negotiation process on air pollution and assessing air quality policies at national/regional level
BMI: Body Mass Index
COPD: Chronic Obstructive Pulmonary Disease
CVD: Cardiovascular diseases
DALY: Disability Adjusted Life Year
DM: Diabetes Mellitus
FARM: Flexible Air Quality Regional Model
HOMA-IR: Homeostatic Model Assessment-Insulin Resistance
IQR: Interquartile range
ISTAT: Italian National Institute of Statistics
MINNI project: agreement between the Italian Ministry of Environment and ENEA
NO_2_: Nitrogen dioxide
O_3_: Ozone
OR: Odds ratio
PM_10_: Particulate matter with a diameter between 2.5 and 10 micrometers (μm)
PM_2.5_: Particulate matter with a diameter of 2.5 micrometers (μm) or less
SD: Standard deviation
T2DM: Type 2 Diabetes Mellitus
VIF: Variance Inflation Factor
VOCs: Volatile Organic Compounds
WHO: World Health Organization

## References

[1] Lim SS, Vos T, Flaxman AD, Danaei G, Shibuya K, Adair-Rohani H, et al A comparative risk assessment of burden of disease and injury attributable to 67 risk factors and risk factor clusters in 21 regions, 1990–2010: A systematic analysis for the Global Burden of Disease Study 2010. Lancet. 2012;380: 2224–2260. doi: 10.1016/S0140-6736(12)61766-8 23245609 10.1016/S0140-6736(12)61766-8 PMC4156511

[2] Bauer M, Moebus S, Mhlenkamp S, Dragano N, Nonnemacher M, Fuchsluger M, et al Urban particulate matter air pollution is associated with subclinical atherosclerosis: Results from the HNR (Heinz Nixdorf Recall) study. J Am Coll Cardiol. 2010;56: 1803–1808. doi: 10.1016/j.jacc.2010.04.065 21087707 10.1016/j.jacc.2010.04.065

[3] Künzli N, Jerrett M, Garcia-Esteban R, Basagaña X, Beckermann B, Gilliland F, et al Ambient air pollution and the progression of atherosclerosis in adults. PLoS One. 2010;5 doi: 10.1371/journal.pone.0009096 20161713 10.1371/journal.pone.0009096 PMC2817007

[4] Bui DS, Burgess JA, Matheson MC, Erbas B, Perret J, Morrison S, et al Ambient wood smoke, traffic pollution and adult asthma prevalence and severity. Respirology. 2013;18: 1101–1107. doi: 10.1111/resp.12108 23627489 10.1111/resp.12108

[5] Kunzli N, Bridevaux P-O, Liu L-JS, Garcia-Esteban R, Schindler C, Gerbase MW, et al Traffic-related air pollution correlates with adult-onset asthma among never-smokers. Thorax. 2009;64: 664–670. doi: 10.1136/thx.2008.110031 19359271 10.1136/thx.2008.110031

[6] Schikowski T, Mills IC, Anderson HR, Cohen A, Hansell A, Kauffmann F, et al Ambient air pollution: A cause of COPD. European Respiratory Journal. 2014 pp. 250–263. doi: 10.1183/09031936.00100112 23471349 10.1183/09031936.00100112

[7] Zanobetti A, Bind M-AC, Schwartz J. Particulate air pollution and survival in a COPD cohort. Environ Heal. 2008;7: 48 doi: 10.1186/1476-069X-7-48 18847462 10.1186/1476-069X-7-48 PMC2572050

[8] Raaschou-Nielsen O, Andersen ZJ, Beelen R, Samoli E, Stafoggia M, Weinmayr G, et al Air pollution and lung cancer incidence in 17 European cohorts: Prospective analyses from the European Study of Cohorts for Air Pollution Effects (ESCAPE). Lancet Oncol. 2013;14: 813–822. doi: 10.1016/S1470-2045(13)70279-1 23849838 10.1016/S1470-2045(13)70279-1

[9] WHO. Global Report on Diabetes. World Heal Organ. 2016;978: 88. doi:ISBN 978 92 4 156525 7

[10] UN General Assembly. Political Declaration of the High-level Meeting of the General Assembly on the Prevention and Control of Non-communicable Diseases. A/RES/66/2. UN New York. 2012;49777: 1–13. doi: 10.1007/BF03038934

[11] Sun Q, Wang A, Jin X, Natanzon A, Duquaine D, Brook RD. Long-term Air Pollution Exposure and Acceleration of Atherosclerosis and Vascular Inflammation in an Animal Model. Jama. 2005;294: 3003–3010. doi: 10.1001/jama.294.23.3003 16414948 10.1001/jama.294.23.3003

[12] Baron AD, Steinberg HO, Chaker H, Leaming R, Johnson A, Brechtel G. Insulin-Mediated Skeletal-Muscle Vasodilation Contributes To Both Insulin Sensitivity and Responsiveness in Lean Humans. J Clin Invest. 1995;96: 786–792. doi: 10.1172/JCI118124 7635973 10.1172/JCI118124 PMC185264

[13] Thomson EM, Pal S, Guénette J, Wade MG, Atlas E, Holloway AC, et al Ozone inhalation provokes glucocorticoid-dependent and independent effects on inflammatory and metabolic pathways. Toxicol Sci. 2016; kfw061. doi: 10.1093/toxsci/kfw061 27037194 10.1093/toxsci/kfw061 PMC12077420

[14] Miller DB, Ghio AJ, Karoly ED, Bell LN, Snow SJ, Madden MC, et al Ozone Exposure Increases Circulating Stress Hormones and Lipid Metabolites in Humans. Am J Respir Crit Care Med. 2016; 1–70. doi: 10.1164/rccm.201508-1599OC 26745856 10.1164/rccm.201508-1599OC PMC5440058

[15] Lockwood AH. Diabetes and air pollution. Diabetes Care. 2002 pp. 1487–1488. 12145265 10.2337/diacare.25.8.1487

[16] Pearson J, Bachireddy C, Shyamprasad S, Goldfine A, Brownstein J. Association between fine particulate matter and diabetes prevalence in the U.S. Diabetes Care. 2010;33: 2196–2201. doi: 10.2337/dc10-0698 20628090 10.2337/dc10-0698 PMC2945160

[17] Solimini AG, D’Addario M, Villari P. Ecological correlation between diabetes hospitalizations and fine particulate matter in Italian provinces. BMC Public Health. BMC Public Health; 2015;15: 708 doi: 10.1186/s12889-015-2018-5 26208978 10.1186/s12889-015-2018-5 PMC4514955

[18] Balti E V, Echouffo-Tcheugui JB, Yako YY, Kengne AP. Air pollution and risk of type 2 diabetes mellitus: A systematic review and meta-analysis. Diabetes Res Clin Pract. Elsevier Ireland Ltd; 2014;106: 161–172. doi: 10.1016/j.diabres.2014.08.010 25262110 10.1016/j.diabres.2014.08.010

[19] Eze IC, Hemkens LG, Bucher HC, Hoffmann B, Schindler C, Kunzli N, et al Association between ambient air pollution and diabetes mellitus in Europe and North America: systematic review and meta-analysis. Env Heal Perspect. 2015;123: 381–389. doi: 10.1289/ehp.1307823 25625876 10.1289/ehp.1307823 PMC4421762

[20] Janghorbani M, Momeni F, Mansourian M. Systematic review and metaanalysis of air pollution exposure and risk of diabetes. Eur J Epidemiol. 2014;29: 231–242. doi: 10.1007/s10654-014-9907-2 24791705 10.1007/s10654-014-9907-2

[21] Jerrett M, Brook R, White LF, Burnett RT, Yu J, Su J, et al Ambient ozone and incident diabetes: A prospective analysis in a large cohort of African American women. Environ Int. Elsevier Ltd; 2017;102: 42–47. doi: 10.1016/j.envint.2016.12.011 28153529 10.1016/j.envint.2016.12.011 PMC5542012

[22] ISTAT. Multipurpose survey on households: health conditions and use of medical services [Internet]. 2013 [cited 23 May 2017]. Available: http://siqual.istat.it/SIQual/visualizza.do?id=0071201

[23] Istat—Multipurpose survey on households: aspects of daily life—general part [Internet]. [cited 23 May 2017]. Available: http://siqual.istat.it/SIQual/visualizza.do?id=0058000

[24] Zanini G, Pignatelli T, Monforti F, Vialetto G, Vitali L, Brusasca G, et al. The MINNI Project: An Integrated Assessment Modeling System For Policy Making. MODSIM 2005 Int Congr Model simulation Model Simul Soc Aust New Zeal. 2005; 2005–2011.

[25] Mircea M, Briganti G, Cappelletti A, Vitali L, Pace G, D’Isidoro M, et al. Ozone simulations with atmospheric modelling system of MINNI project: A multi year evaluation over Italy. HARMO 2011—Proc 14th Int Conf Harmon within Atmos Dispers Model Regul Purp. 2011; 52–56.

[26] Silibello C, Calori G, Brusasca G, Giudici A, Angelino E, Fossati G, et al Modelling of PM10 concentrations over Milano urban area using two aerosol modules. Environ Model Softw. 2008;23: 333–343. doi: 10.1016/j.envsoft.2007.04.002

[27] Gariazzo C, Papaleo V, Pelliccioni A, Calori G, Radice P, Tinarelli G. Application of a Lagrangian particle model to assess the impact of harbour, industrial and urban activities on air quality in the Taranto area, Italy. Atmos Environ. 2007;41: 6432–6444. doi: 10.1016/j.atmosenv.2007.06.005

[28] Piersanti A, Righini G, Cremona G, Ciancarella L, Cionni I, D’Isidoro M, et al. GIS-based procedure for evaluation of performances of the Italian atmospheric modelling system simulated data versus observed measurements. 6th Bienn Meet Int Environ Model Softw Soc Manag Resour a Ltd Planet, iEMSs 2012. 2012; 1375–1382. Available: http://www.scopus.com/inward/record.url?eid=2-s2.0-84894165478&partnerID=40&md5=a0b95afdbe7800944f6065685cfa926f

[29] Mircea M, Ciancarella L, Briganti G, Calori G, Cappelletti A, Cionni I, et al Assessment of the AMS-MINNI system capabilities to simulate air quality over Italy for the calendar year 2005. Atmos Environ. Elsevier Ltd; 2014;84: 178–188. doi: 10.1016/j.atmosenv.2013.11.006

[30] WHO. WHO Air quality guidelines for particulate matter, ozone, nitrogen dioxide and sulfur dioxide: global update 2005: summary of risk assessment World Heal Organ 2006; 1–22. doi: 10.1016/0004-6981(88)90109-6

[31] ISTAT. EPICENTRO—National Diabetes Fact Sheet [Internet]. 2015 [cited 23 May 2017]. Available: http://www.epicentro.iss.it/igea/en/DiabetesFactSheet.asp#3

[32] Chen H, Burnett RT, Kwong JC, Villeneuve PJ, Goldberg MS, Brook RD, et al Risk of incident diabetes in relation to long-term exposure to fine particulate matter in Ontario, Canada. Environ Health Perspect. 2013;121: 804–810. doi: 10.1289/ehp.1205958 [Online 23 December 2010]; Hotamisligil, G.S., Inflammation and metabolic disorders (2006) Nature, 444, pp. 860–867; Hu, F.B., Manson, J.A.E., Stampfer, M.J., Colditz, G., Liu, S., Solomon, C.G., Diet, lifestyle, and the risk of type 2 diabetes mellitus in women (2001) N Engl J Med, 345, pp. 790–797; Hux, J.E., Ivis, F., Flintoft, V., Bica, A., Determination of prevalence and incidence using a validated administrative data algorithm (2002) Diabetes C 23632126 10.1289/ehp.1205958 PMC3701997

[33] Eze IC, Schaffner E, Fischer E, Schikowski T, Adam M, Imboden M, et al Long-term air pollution exposure and diabetes in a population-based Swiss cohort. Environ Int. 2014;70: 95–105. doi: 10.1016/j.envint.2014.05.014 24912113 10.1016/j.envint.2014.05.014

[34] Dijkema MBA, Mallant SF, Gehring U, van den Hurk K, Alssema M, van Strien RT, et al Long-term exposure to traffic-related air pollution and type 2 diabetes prevalence in a cross-sectional screening-study in the Netherlands. Environ Health. BioMed Central Ltd; 2011;10: 76 doi: 10.1186/1476-069X-10-76 21888674 10.1186/1476-069X-10-76 PMC3200985

[35] Rao X, Montresor-Lopez J, Puett R, Rajagopalan S, Brook RD. Ambient air pollution: an emerging risk factor for diabetes mellitus. Curr Diab Rep. 2015;15: 603 doi: 10.1007/s11892-015-0603-8 25894943 10.1007/s11892-015-0603-8

[36] Coogan PF, White LF, Jerrett M, Brook RD, Su JG, Seto E, et al Air pollution and incidence of hypertension and diabetes mellitus in black women living in Los Angeles. Circulation. 2012;125: 767–772. doi: 10.1161/CIRCULATIONAHA.111.052753 22219348 10.1161/CIRCULATIONAHA.111.052753 PMC3326581

[37] Weinmayr G, Hennig F, Fuks K, Nonnemacher M, Jakobs H, Möhlenkamp S, et al Long-term exposure to fine particulate matter and incidence of type 2 diabetes mellitus in a cohort study: effects of total and traffic-specific air pollution. Environ Heal. 2015;14: 53 doi: 10.1186/s12940-015-0031-x 26087770 10.1186/s12940-015-0031-x PMC4479324

[38] Brook RD, Jerrett M, Brook JR, Bard RL, Finkelstein MM. The Relationship Between Diabetes Mellitus and Traffic-Related Air Pollution. J Occup Environ Med. 2008;50: 32–38. doi: 10.1097/JOM.0b013e31815dba70 18188079 10.1097/JOM.0b013e31815dba70

[39] Krämer U, Herder C, Sugiri D, Strassburger K, Schikowski T, Ranft U, et al Traffic-related air pollution and incident type 2 diabetes: Results from the SALIA cohort study. Environ Health Perspect. 2010;118: 1273–1279. doi: 10.1289/ehp.0901689 20504758 10.1289/ehp.0901689 PMC2944089

[40] Andersen ZJ, Raaschou-Nielsen O, Ketzel M, Jensen SS, Hvidberg M, Loft S, et al Diabetes incidence and long-term exposure to air pollution: A cohort study. Diabetes Care. 2012;35: 92–98. doi: 10.2337/dc11-1155 22074722 10.2337/dc11-1155 PMC3241311

[41] Hansen AB, Ravnskjær L, Loft S, Andersen KK, Bräuner EV, Baastrup R, et al Long-term exposure to fine particulate matter and incidence of diabetes in the Danish Nurse Cohort. Environ Int. The Authors; 2016;91: 243–250. doi: 10.1016/j.envint.2016.02.036 26989812 10.1016/j.envint.2016.02.036

[42] Puett RC, Hart JE, Schwartz J, Hu FB, Liese AD, Laden F. Are particulate matter exposures associated with risk of type 2 diabetes? Environ Health Perspect. 2011;119: 384–389. doi: 10.1289/ehp.1002344 21118784 10.1289/ehp.1002344 PMC3060003

[43] Geiss LS, Wang J, Cheng YJ, Thompson TJ, Barker L, Li Y, et al Prevalence and Incidence Trends for Diagnosed Diabetes Among Adults Aged 20 to 79 Years, United States, 1980–2012. Jama. 2014;312: 1218 doi: 10.1001/jama.2014.11494 25247518 10.1001/jama.2014.11494

[44] Miller KA, Siscovick DS, Sheppard L, Shepherd K, Sullivan JH, Anderson GL, et al Long-Term Exposure to Air Pollution and Incidence of Cardiovascular Events in Women. N Engl J Med. 2007;356: 447–458. doi: 10.1056/NEJMoa054409 17267905 10.1056/NEJMoa054409

[45] Beelen R, Raaschou-Nielsen O, Stafoggia M, Andersen ZJ, Weinmayr G, Hoffmann B, et al Effects of long-term exposure to air pollution on natural-cause mortality: An analysis of 22 European cohorts within the multicentre ESCAPE project. Lancet. 2014;383: 785–795. doi: 10.1016/S0140-6736(13)62158-3 24332274 10.1016/S0140-6736(13)62158-3

[46] Mills NL, T??rnqvist H, Robinson SD, Gonzalez M, Darnley K, MacNee W, et al Diesel exhaust inhalation causes vascular dysfunction and impaired endogenous fibrinolysis. Circulation. 2005;112: 3930–3936. doi: 10.1161/CIRCULATIONAHA.105.588962 16365212 10.1161/CIRCULATIONAHA.105.588962

[47] Kelishadi R, Mirghaffari N, Poursafa P, Gidding SS. Lifestyle and environmental factors associated with inflammation, oxidative stress and insulin resistance in children. Atherosclerosis. 2009;203: 311–319. doi: 10.1016/j.atherosclerosis.2008.06.022 18692848 10.1016/j.atherosclerosis.2008.06.022

[48] Sun Q, Yue P, Deiuliis JA, Lumeng CN, Kampfrath T, Mikolaj MB, et al Ambient air pollution exaggerates adipose inflammation and insulin resistance in a mouse model of diet-induced obesity. Hear Lung. 2009;119: 538–546. doi: 10.1161/CIRCULATIONAHA.108.799015.Ambient 10.1161/CIRCULATIONAHA.108.799015 PMC3845676 19153269

[49] Eze IC, Imboden M, Kumar A, Adam M, von Eckardstein A, Stolz D, et al A common functional variant on the pro-inflammatory Interleukin-6 gene may modify the association between long-term PM10 exposure and diabetes. Environ Heal. Environmental Health; 2016;15: 39 doi: 10.1186/s12940-016-0120-5 26911440 10.1186/s12940-016-0120-5 PMC4765217

[50] Rajagopalan S, Brook RD. Air pollution and type 2 diabetes: Mechanistic insights. Diabetes. 2012;61: 3037–3045. doi: 10.2337/db12-0190 23172950 10.2337/db12-0190 PMC3501850

[51] Dubowsky SD, Suh H, Schwartz J, Coull BA, Gold DR. Diabetes, Obesity, and Hypertension May Enhance Associations between Air Pollution and Markers of Systemic Inflammation. Environ Health Perspect. 2006;114: 992–998. doi: 10.1289/ehp.8469 16835049 10.1289/ehp.8469 PMC1513328

[52] Bass V, Gordon CJ, Jarema KA, MacPhail RC, Cascio WE, Phillips PM, et al Ozone induces glucose intolerance and systemic metabolic effects in young and aged brown Norway rats. Toxicol Appl Pharmacol. 2013;273: 551–560. doi: 10.1016/j.taap.2013.09.029 24103449 10.1016/j.taap.2013.09.029 PMC4343260

[53] Sullivan JH, Hubbard R, Liu SL-J, Shepherd K, Trenga CA, Koenig JQ, et al A community study of the effect of particulate matter on blood measures of inflammation and thrombosis in an elderly population. Environ Heal. 2007;6: 3 doi: 10.1186/1476-069X-6-3 17270049 10.1186/1476-069X-6-3 PMC1800891

[54] Steinvil A, Kordova-Biezuner L, Shapira I, Berliner S, Rogowski O. Short-term exposure to air pollution and inflammation-sensitive biomarkers. Environ Res. 2008;106: 51–61. doi: 10.1016/j.envres.2007.08.006 17915210 10.1016/j.envres.2007.08.006

[55] American Diabetes Association. Diagnosis and Classification of Diabetes Mellitus. Diabetes Care. 2012;35: S64–S71. doi: 10.2337/dc12-s064 22187472 10.2337/dc12-s064 PMC3632174

