# Supplementary figures and images for: Association between PM10, PM2.5, NO2, O3 and self-reported diabetes in Italy: A cross-sectional, ecological study
Source: PLoS One. 2018 Jan 17;13(1):e0191112. doi: 10.1371/journal.pone.0191112 (PMC5771616; doi:10.1371/journal.pone.0191112)

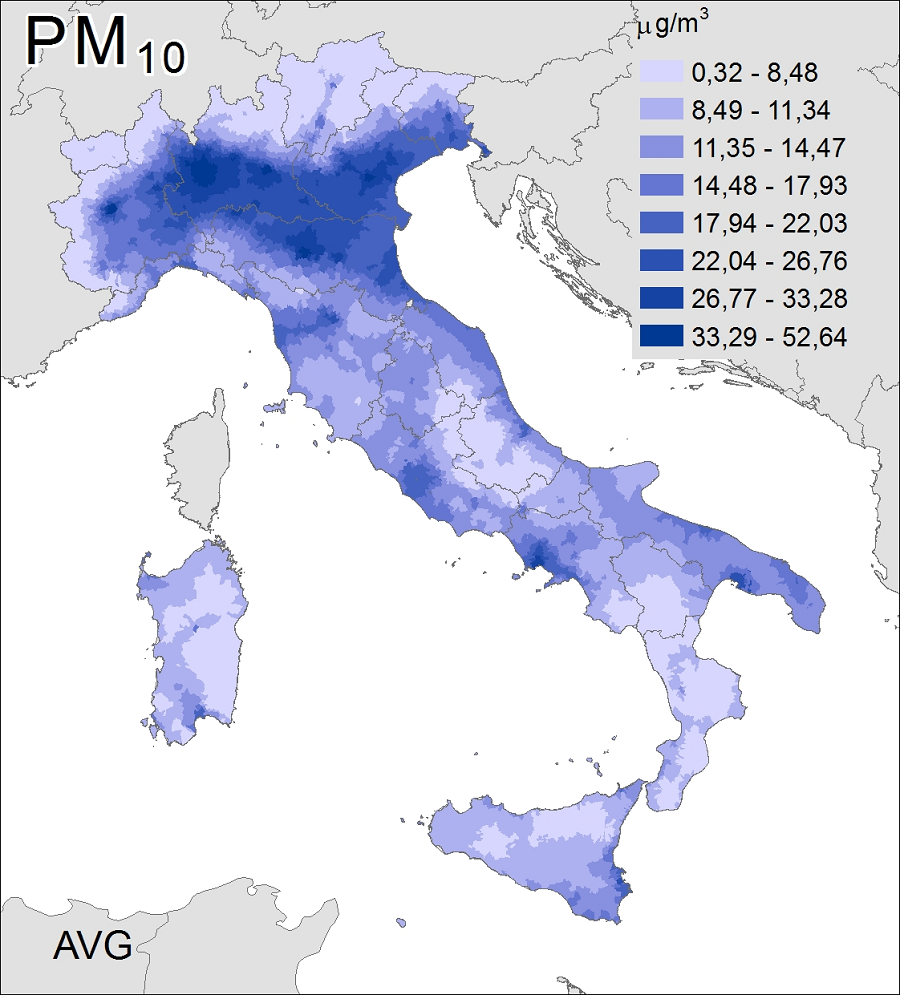

Supplement: S1 Fig — Averaged annual levels for years 2003, 2005, 2007 and 2010. (TIF) [file pone.0191112.s001.tif]

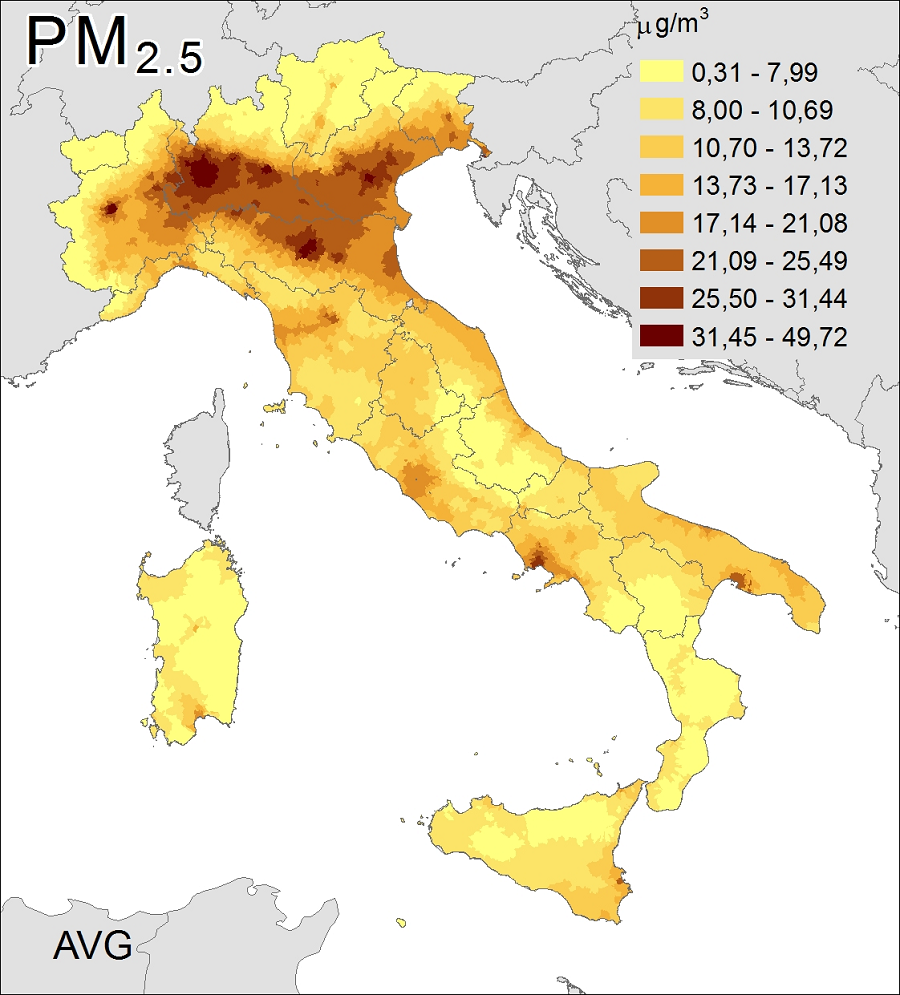

Supplement: S2 Fig — Averaged annual levels for years 2003, 2005, 2007 and 2010. (TIF) [file pone.0191112.s002.tif]

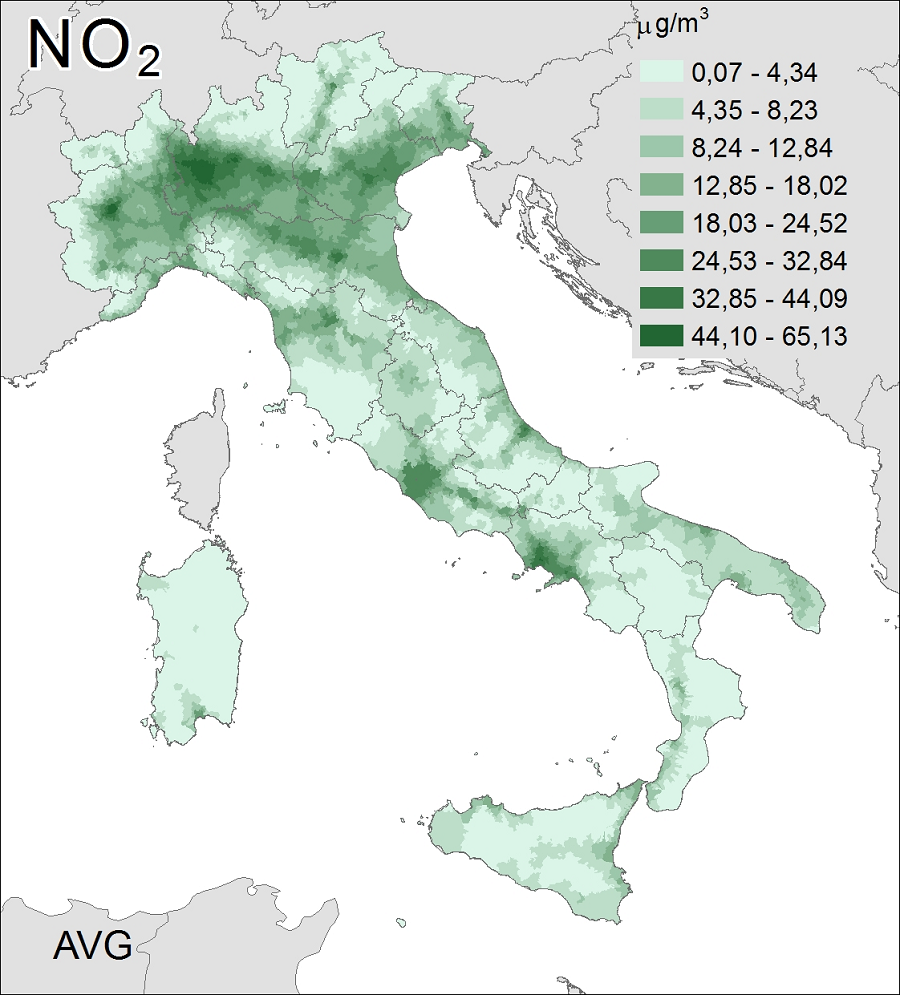

Supplement: S3 Fig — Averaged annual levels for years 2003, 2005, 2007 and 2010. (TIF) [file pone.0191112.s003.tif]

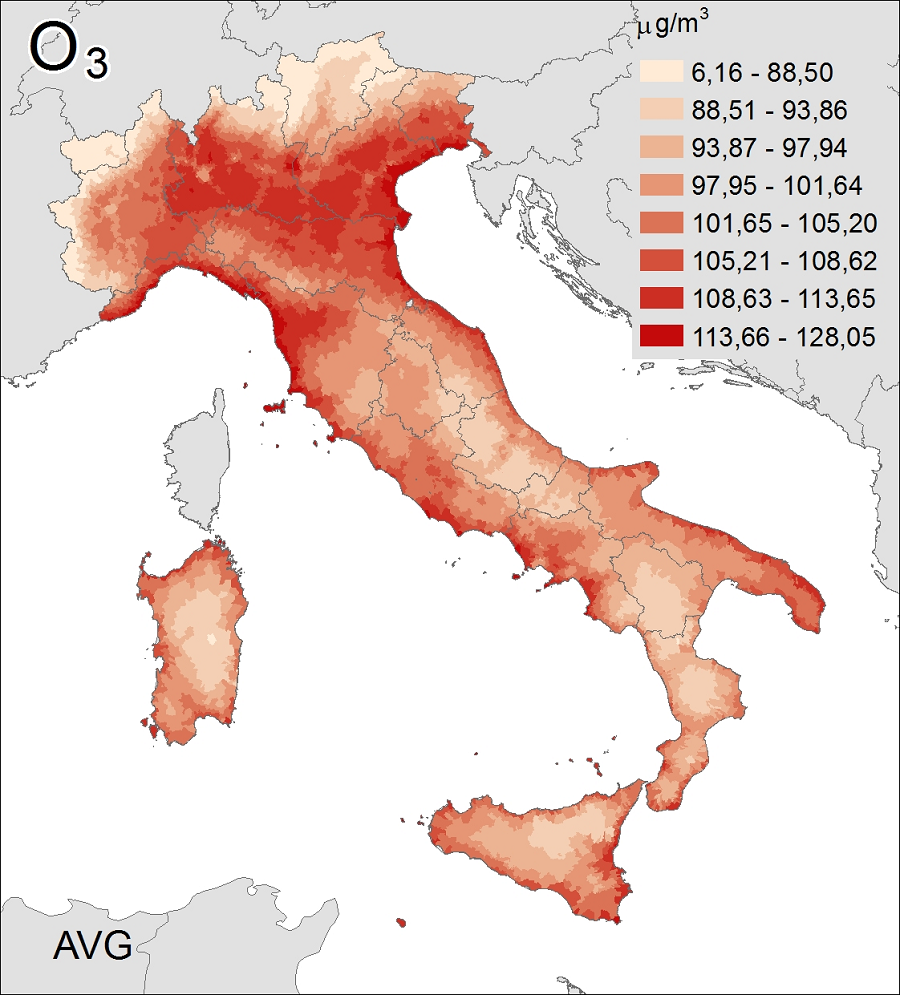

Supplement: S4 Fig — Averaged annual levels for years 2003, 2005, 2007 and 2010. (TIF) [file pone.0191112.s004.tif]
