# Supplementary material for: Association between PM10, PM2.5, NO2, O3 and self-reported diabetes in Italy: A cross-sectional, ecological study
Source: PLoS One. 2018 Jan 17;13(1):e0191112. doi: 10.1371/journal.pone.0191112 (PMC5771616; doi:10.1371/journal.pone.0191112)
Supplement: S1 Table — Mixed models with region and survey year as random components. AIC: Akaike Information Criterion. *from likelihood ratio test, each model tested with the preceding one. (DOCX) [file pone.0191112.s005.docx]

**S1 Table.**

| Model | Predictors | AIC | p-value* |
| --- | --- | --- | --- |
| M1 | CVD | 221,863 | / |
| M2 | M1 + BMI | 221,797 | <0.001 |
| M3 | M2 + age | 214,571 | <0.001 |
| M4 | M3 + occupational status | 213,799 | <0.001 |
| M5 | M4 + household income | 213,348 | <0.001 |
| M6 | M5 + educational level | 213,007 | <0.001 |
| M7 | M6 + physical activity | 212,828 | <0.001 |
| M8 | M7 + sex | 212,612 | <0.001 |
| M9 | M8 + smoking status | 212,539 | <0.001 |
| M10 | M9 + marital status | 212,491 | <0.001 |
| M11 | M10 + PM10 | 212,475 | 0,002 |
| M12 | M10 + PM2.5 | 212,475 | 0,002 |
| M13 | M10 + NO2 | 212,470 | <0.001 |
| M14 | M10 + O3 | 212,477 | 0,007 |
